# Risk Factors for Hyperosmolar Hyperglycemic State in Pediatric Type 2 Diabetes

**DOI:** 10.1155/2023/1318136

**Published:** 2023-06-09

**Authors:** Estelle M. Everett, Timothy Copeland, Lauren E. Wisk, Lily C. Chao

**Affiliations:** ^1^Division of Endocrinology, Diabetes, & Metabolism, Department of Medicine, David Geffen School of Medicine, University of California, Los Angeles, CA, USA; ^2^Division of General Internal Medicine & Health Services Research, Department of Medicine, David Geffen School of Medicine, University of California, Los Angeles, CA, USA; ^3^VA Greater Los Angeles Healthcare System, Los Angeles, CA, USA; ^4^Department of Health Policy and Management, Fielding School of Public Health, University of California, Los Angeles, CA, USA; ^5^Center for Endocrinology, Diabetes and Metabolism, Children's Hospital Los Angeles, Los Angeles, CA, USA; ^6^Department of Pediatrics, Keck School of Medicine, University of Southern California, Los Angeles, CA, USA

## Abstract

**Background:**

There is a paucity of data on the risk factors for the hyperosmolar hyperglycemic state (HHS) compared with diabetic ketoacidosis (DKA) in pediatric type 2 diabetes (T2D).

**Methods:**

We used the national Kids' Inpatient Database to identify pediatric admissions for DKA and HHS among those with T2D in the years 2006, 2009, 2012, and 2019. Admissions were identified using ICD codes. Those aged <9yo were excluded. We used descriptive statistics to summarize baseline characteristics and Chi-squared test and logistic regression to evaluate factors associated with admission for HHS compared with DKA in unadjusted and adjusted models.

**Results:**

We found 8,961 admissions for hyperglycemic emergencies in youth with T2D, of which 6% were due to HHS and 94% were for DKA. These admissions occurred mostly in youth 17–20 years old (64%) who were non-White (Black 31%, Hispanic 20%), with public insurance (49%) and from the lowest income quartile (42%). In adjusted models, there were increased odds for HHS compared to DKA in males (OR 1.77, 95% CI 1.42–2.21) and those of Black race compared to those of White race (OR 1.81, 95% CI 1.34–2.44). Admissions for HHS had 11.3-fold higher odds for major or extreme severity of illness and 5.0-fold higher odds for mortality.

**Conclusion:**

While DKA represents the most admissions for hyperglycemic emergencies among pediatric T2D, those admitted for HHS had higher severity of illness and mortality. Male gender and Black race were associated with HHS admission compared to DKA. Additional studies are needed to understand the drivers of these risk factors.

## 1. Introduction

Diabetic ketoacidosis (DKA) and hyperglycemic hyperosmolar state (HHS) are hyperglycemic emergencies that can occur in both type 1 and type 2 diabetes in the setting of insulin deficiency. In both conditions, hyperglycemia contributes to osmotic diuresis, dehydration, and electrolyte abnormalities. It is thought that the relative state of insulin deficiency contributes to the differing clinical presentations. DKA occurs in the state of profound insulin deficiency, resulting in the lack of suppression of gluconeogenesis, increased glycogenolysis and lipolysis, and decreased glucose utilization. Increased lipolysis results in the conversion of free fatty acids to ketones including beta-hydroxybutyrate, acetone, and acetoacetate [1]. The ensuing acidemia and acute symptoms of nausea, vomiting, and abdominal pain prompt patients and families to seek emergent medical care. In HHS, while insulin deficiency is present, there is still sufficient insulin production to suppress gluconeogenesis and prevent lipolysis [2]. Dehydration is more profound in HHS, and severe electrolyte abnormalities can occur. Hyperosmolarity, a consequence of severe dehydration and hyperglycemia, results to maintain circulatory volume. While reversal of ketoacidosis with insulin infusion is the mainstay of DKA management, volume repletion and gradual correction of hyperglycemia are essential to prevent vascular collapse in HHS [3]. As the clinical presentation of DKA and HHS can be similar, biochemical criteria are used to distinguish between these two entities. In HHS, venous pH is above 7.25 (arterial pH > 7.3), serum bicarbonate is above 15 mmol/L, plasma glucose concentration is above 600 mg/dL, ketones are absent or small in quantity, and an effective (not measured) serum osmolarity is above 320 mOsm/kg [4].

Although more common in type 1 diabetes, hyperglycemic emergencies are also observed in type 2 diabetes in children [5]. Despite the rising prevalence of pediatric type 2 diabetes in the U.S., the SEARCH registry reported a decline in DKA incidence at the presentation from 2002-2003 to 2008–2010 [6]. However, during the COVID-19 pandemic, higher DKA and HHS incidence was observed in new-onset type 2 diabetes in youth [7–9]. Given the historically low incidence of hyperglycemic emergencies in children with type 2 diabetes, there is a paucity of data on the demographics and risk factors for children with these conditions. Studies on HHS in children are largely descriptive, single-center case reports that represent a homogenous patient population [10–12]. It is also unknown if the demographics differ between DKA and HHS in youth with type 2 diabetes. We present here the first epidemiological evaluation of hyperglycemic emergencies among pediatric type 2 diabetes, comparing relative risk factors, resource utilization, and mortality rates for DKA and HHS.

## 2. Methods

### 2.1. Study Population

We used the Healthcare Cost and Utilization Project (HCUP) Kids' Inpatient Database (KID), developed by the AHRQ [13]. KID is a publicly available deidentified database containing admissions from children and youth ≤20 years old (yo) from 42,000 hospitals across 46 states, sampled at a rate of 80% for non-newborn admissions. KID data are available every 3 years, but 2015 data were not released, and instead, 2016 data were released due to the transition from the International Code of Diseases (ICD) 9 to ICD-10 coding. We identified patients admitted with a diagnosis of type 2 diabetes and DKA in study years 2006, 2009, 2012, 2016, and 2019 using the ICD-9 and ICD-10 codes for type 2 diabetes with ketoacidosis (250.10, 250.12, E11.10, E11.11) and type 2 diabetes with hyperosmolality (250.20, 250.22, E11.00, E11.01). However, the ICD-9 codes for DKA in type 2 diabetes were discontinued on September 30, 2015, and the corollary ICD-10 did not go into effect until 10/1/2017. We thus excluded admissions occurring in 2016. We also excluded admissions with concomitant type 1 diabetes ICD-9/10 codes and admission of those aged less than 9 years old as they are unlikely to have a true diagnosis of type 2 diabetes [14]. Although type 2 diabetes is reported in children younger than the age of 10, they represent a minority of the patient population [5]. As we do not have access to individual records to verify the coding accuracy, which is especially a concern in the age category of 5 to 8 years, we have elected to exclude their data in this analysis as well.

### 2.2. Data Elements

We described patient levels and hospital factors associated with both DKA and HHS admissions. These data elements were described in a former publication [15]. Patient level factors included age, biological sex, self-reported race and ethnicity, insurance payer, urban/rural status, and median household income. Admission payers included public insurance (e.g., Medicare, Medicaid), private payers (e.g., private HMOs, PPOs), other (worker's compensation, title V, Civilian Health and Medical Health and Medical Program of the Department of Veteran Affairs or other government programs), and self-pay or no charge (e.g., charity care). Median household income was reported as quartiles and was determined based on median household income per zip code for the calendar year. Patient location was reported per the 2010 United States urban-rural classification, which defines an urbanized area as 50,000 residents or more and a nonurban area as less than 50,000 residents. Hospital-level variables included hospital region, ownership, and size. The severity of illness on admission was determined using All Patient Refined Diagnosis-Related Groups (APR-DRG), which classify patients according to their reasons for admission, severity of illness, risk of mortality, and resource intensity. Length of stay (LOS) is defined as the difference in days between the admission date and the discharge date. Total charges for admission are reported from the hospital to KID and do not include professional fees and noncovered charges. Charges accounted for inflation in the Consumer Price Index and were reported as 2019 US dollars.

### 2.3. Statistical Analysis

Weights provided by KID were used to generate nationally representative estimates of hospital admissions, and all analyses accounted for the stratified sampling design. We used descriptive statistics to summarize the characteristics of all hyperglycemic emergency admissions and those of DKA and HHS admissions. Counts less than or equal to 10 are noted as ≤10 per HCUP policy to preserve the amenity and privacy rights of those individuals. Chi-squared test was used to evaluate for unadjusted statistical differences between DKA and HHS admissions. We performed logistic regression to evaluate what patient and hospital factors were associated with HHS admission in an adjusted model. We evaluated for a difference in clinical features at presentation and discharge, specifically the severity of illness on admission and mortality. We performed logistic regression to evaluate the adjusted odds of a major or extreme severity of illness admission and of mortality in HHS compared with DKA. All analyses were performed with Stata version 15.1 (StataCorp, College Station, TX). This study was IRB-exempt as it was a secondary analysis of pre-existing and deidentified data. This report follows the STROBE reporting guidelines for cross-sectional studies [16].

## 3. Results

We found 8,961 admissions for hyperglycemic emergencies in youth with T2D, of which 6% were due to HHS and 94% were for DKA (Table 1). These admissions occurred mostly in youth 17–20 years old (64%) who were non-White race (Black 31%, Hispanic 20%) with public insurance (49%) and from the lowest income quartile (42%) and most admitted in large (60%) private (69%) hospitals and in Southern United States (49%).

When HHS admissions were compared to DKA admissions, we found that HHS admissions were most likely to occur in males (OR 1.77, 95% CI 1.42–2.21, *p* < 0.001) and those of Black race (OR 1.81, 95% CI 1.34–2.44, *p* < 0.001) compared to those of White race (Table 2). There was no significant difference between those admitted for HHS compared to DKA with regard to age, other racial-ethnic groups, insurance payers, income, hospital region, or ownership. Small hospitals had lower odds of HHS admission (OR 0.61, 95% CI 0.41–0.89, *p*=0.01) compared to large hospitals.

Admissions for HHS had higher severity of illness, with 55% classified as major and 13% as extreme severity of illness, whereas for DKA, 12% were classified as major and 3% as extreme severity of illness (Table 3). HHS admissions had an 11.3-fold increase in odds (OR 11.3, 95% CI 8.9–14.4) for classification as major or extreme severity of illness, after adjusting for patient and hospital features (data not shown). The mortality rate was 1.8% for HHS compared to 0.3% in DKA. HHS admissions had a 5-fold increased adjusted odds for mortality (OR 4.97, 95% CI 2.1–11.9) compared with DKA (data not shown).

In terms of LOS and health expenditures, HHS admissions were associated with a slightly higher LOS of 3.94 (95% CI 3.12–4.75) compared to 3.16 days (95% CI 3.07–3.26) in DKA (Table 4). Similarly, the admission cost was higher at $35,034 (95% CI $26,616–$43,453) in HHS compared to $27,008 ($25,846–$28,170) in DKA.

## 4. Discussion

We present here the first epidemiological analysis of DKA and HHS in children and youth with type 2 diabetes in a nationally representative cohort. Using the Kids' Inpatient Database, that consists of admissions data from 42,000 hospitals across 46 states in the United States, we identified 8,961 cases of hyperglycemic emergencies in patients between ages 9 to 20. DKA and HHS occurred mostly in the 17-to-20-year age group although the mean age for DKA was slightly younger (16.9 yo [95% CI 16.8–17.0] vs. 17.5 yo [95% CI 17.2–17.8]). Compared to DKA admissions, we found that male sex and Black race are independent risk factors for HHS admissions. Patients with HHS had an increased severity of illness and length of stay. In this large cohort of HHS patients (560 cases), the mortality rate was 1.8%, representing a 5.0-fold increased adjusted odds compared with DKA. This mortality rate was lower than the 3.3% previously reported for pediatric HHS during the years 1997–2009 [17]. This may be due to the overall rise of pediatric type 2 diabetes cases and increased recognition of these two distinct hyperglycemic emergencies, which may facilitate more appropriate treatment plans to reduce morbidity and mortality.

We report here a disproportionate representation of males in HHS compared to DKA. Pediatric type 2 diabetes is female-predominant in the U.S., with the female-to-male ratio ranging from 1.2 to 1.8, with the lowest ratio coming from a retrospective review of new-onset type 2 diabetes cases and higher ratios coming from a prospective clinical trial or natural history registries [7, 18, 19]. We found that male accounts for 62.0% of all HHS cases. Given the infrequent occurrence of HHS in pediatric type 2 diabetes, most reports on this condition consist of small case series or retrospective reviews consisting of 7 to 9 cases [5, 10, 11]. In both Fourtner et al. and Morales et al., male sex was observed in 71 to 85% of HHS cases [10, 11]. Bagdure et al. previously analyzed the epidemiology of HHS syndrome in children aged 0 to 17 between the years 1997 to 2009 in the U.S. and similarly found that male comprised of 60.2% of T2D patients with HHS admissions (317 patients) [17]. Analyses in adult T2D HHS cases have not shown a male predominance [20, 21]. The cause of male predominance in pediatric HHS cases is unknown. Several reports have shown reduced healthcare usage in older adolescent males (aged 16 to 20 years) and preventive health services in young adult males [22–24]. A delay or omission of routine physical examination and screening services may delay the diagnosis of type 2 diabetes in at-risk youth. A delay in accessing medical care may also contribute to the higher severity of illness of HHS admissions. The signs and symptoms of HHS are more insidious compared to DKA, which may delay clinical presentation until there are severe metabolic decompensation and symptoms of weakness and altered mental status, which may contribute to the higher severity of illness at presentation. Future studies evaluating the relationship between routine preventive care and HHS as well as barriers in seeking care in older male adolescents may provide more insight into this finding.

We also identified Black race as a risk factor for HHS hospitalization among pediatric type 2 diabetes admitted for hyperglycemic emergencies. Previous case reports have highlighted a disproportionate representation of Black youth in HHS [10]. In a 6-year retrospective review of HHS cases in 10- to 30-year-olds, Canarie et al. also found that 77% of patients with presumed type 2 diabetes were African American [25]. Other studies focused on adults with type 2 diabetes have also shown that Black race was a risk factor for the hyperglycemic crisis, even after adjusting for socioeconomic, clinical, and treatment-related factors [26]. Racial differences in *β*-cell glucose response may contribute to the increased propensity for HHS among Black youth. A study of healthy adolescents showed that insulin secretory response was higher in Blacks compared to Whites in hyperglycemic clamp studies [27]. Compared to White adolescents, obese Black adolescents exhibited an early insulin secretory response and decreased insulin clearance during a 2-hour oral glucose tolerance test [28]. Reduced insulin clearance in Black adolescents in the Bogalusa heart study has also been reported previously [29]. It is possible that the enhanced *β*-cell glucose response in Black youth may protect them from severe insulin deficiency but increase their risk for HHS if diabetes remains undiagnosed. HHS may also occur due to barriers in accessing medical care. Social factors such as institutional racism, implicit clinician bias, and lower quality of care may foment distrust in the healthcare system and a reluctance to seek medical care. Historical discriminatory housing policies have also contributed to racially segregated neighborhoods with inadequate housing, food swamps, and exposure to environmental pollutants, which all contribute to the higher risk for type 2 diabetes [30, 31]. The development of strategies to improve equitable healthcare access and culturally sensitive care models has the potential for early detection of type 2 diabetes, which in turn may reduce hyperglycemic emergencies and associated morbidity and mortality in this vulnerable population.

The limitations of this study include potential misclassification of diabetes type, DKA or HHS diagnoses, given KID is a claims-based database. In our analysis, 98 admissions (1.5%) had ICD codes for both DKA and HHS, which may represent hyperosmolar DKA. As this assumption could not be verified, these cases were removed from this analysis. In addition, we are unable to account for changes in coding practices in the setting of ICD-9 and ICD-10 transition. During this transition, no ICD code was available to capture DKA in type 2 diabetes in 2016, precluding us from calculating DKA case rates over time. Compared to 2006, the odds ratio of HHS to DKA admission rose in 2009 and 2012, however, but was unchanged as of 2019 (Table 2). Although it is not possible to determine causation from this analysis, we speculate that increased awareness of the distinction between these two entities may have improved clinical outcomes. Another limitation is that this dataset does not allow us to distinguish between new onset diabetes cases from repeat admissions. Finally, as this is an inpatient database, we are unable to report the total prevalence of type 2 diabetes to calculate the incidence and prevalence of HHS and DKA. Nevertheless, this study is one of the largest studies that capture the most recent estimates of DKA and HHS in pediatric type 2 diabetes in a real world, nationally representative sample. We identified distinct patient risk factors for DKA and HHS, which speaks to the complex interaction between the pathophysiology of these hyperglycemic states and race and sex.

With the rise in pediatric type 2 diabetes cases, hyperglycemic emergencies will become more prevalent. Strategies that identify at-risk populations have the potential to prevent DKA and HHS. These strategies may include increased screening for type 2 diabetes and improving access to medical care for the at-risk population. Given the increased disease severity and mortality of HHS, there is a pressing need for not only endocrinologists but also emergency medicine physicians to recognize the distinction in presentation and treatment between DKA and HHS. Future studies are also needed to study the prevalence, disease severity, and mortality of hyperosmolar DKA. Current guidelines for hyperosmolar DKA and HHS are adapted from adult practice guidelines. However, the lack of consensus on optimal fluid rates in children with obesity and concern for cerebral edema remain treatment conundrums. A multicenter clinical trial is needed to provide evidence-based treatment for fluid and insulin infusion rates for children with hyperosmolar DKA and HHS, to reduce the morbidity and mortality associated with these hyperglycemic crises.

## Figures and Tables

**Table 1:** Admission characteristics for hyperglycemic emergencies in pediatric type 2 diabetes.

	HHS		DKA		*P* value	Total	
	*N*	(%)^*∗*^	*N*	(%)^*∗*^		*N*	(%)^∗^
Total	560	6.2	8,401	93.8		8,961	100.0
Age					0.010		
9–12	39	7.0	1,075	12.8		1,114	12.4
13–16	123	21.9	1,929	23.0		2,052	22.9
17–20	397	70.8	5,358	63.8		5,754	64.2
Unknown	≤10	0.3	40	0.5		42	0.5
Sex					<0.001		
Male	346	61.9	4,144	49.3		4,491	50.1
Female	211	37.6	4,222	50.3		4,433	49.5
Unknown	≤10	0.5	35	0.4		38	0.4
Race/ethnicity					<0.001		
White	129	23.0	2,540	30.2		2,669	29.8
Black	246	43.9	2,613	31.1		2,859	31.9
Hispanic	95	17.0	1,716	20.4		1,811	20.2
Asian or Pacific Islander	≤10	1.8	127	1.5		137	1.5
Native American	≤10	1.5	100	1.2		109	1.2
Other/unknown	72	12.9	1,305	15.5		1,378	15.4
Payer					0.621		
Private	164	29.3	2,684	32.0		2,848	31.8
Public	282	50.3	4,151	49.4		4,433	49.5
Self-pay or no charge	88.0	15.8	1,168	13.9		1,256	14.0
Other/unknown	26	4.6	398	4.7		423	4.7
Urbanicity					0.102		
Nonurban areas	85	15.2	1,434	17.1		1,519	17.0
Urban areas	468	83.5	6,924	82.4		7,392	82.5
Unknown	≤10	1.3	43	0.5		51	0.6
Household income					0.842		
Quartile 1	235	42.0	3,540	42.1		3,775	42.1
Quartile 2	141	25.1	2,090	24.9		2,231	24.9
Quartile 3	106	18.9	1,603	19.1		1,709	19.1
Quartile 4	59	10.6	965	11.5		1,025	11.4
Unknown	19	3.4	204	2.4		222	2.5
Region of hospital					0.261		
Northeast	86	15.4	1,192	14.2		1,278	14.3
Midwest	104	18.6	1,542	18.4		1,646	18.4
South	286	51.1	4,059	48.3		4,345	48.5
West	83	14.9	1,609	19.2		1,693	18.9
Ownership					0.001		
Public	128	22.8	2,643	31.5		2,771	30.9
Private	432	77.2	5,759	68.5		6,191	69.1
Bed size					0.030		
Small	54	9.6	1,313	15.6		1,367	15.3
Medium	135	24.1	1,846	22.0		1,981	22.1
Large	353	63.0	5,038	60.0		5,390	60.2
Unknown	18	3.3	205	2.4		223	2.5
Year					<0.001		
2006	107	19.1	2,423	28.8		2,530	28.2
2009	148	26.4	1,759	20.9		1,907	21.3
2012	160	28.5	1,490	17.7		1,650	18.4
2019	145	26.0	2,729	32.5		2,875	32.1

HHS = hyperosmolar hyperglycemic state, DKA = diabetic ketoacidosis, ^*∗*^percentage of all admissions.

**Table 2:** Odds for HHS admission compared to DKA admission.

	OR	95% CI		*P* value
Age				
9–12		Reference		
13–16	1.81	1.11	2.93	0.017
17–20	2.12	1.35	3.32	0.001
Unknown	0.72	0.04	14.67	0.831
Sex				
Male	1.77	1.42	2.21	<0.001
Female		Reference		
Unknown	5.50	0.61	49.64	0.129
Race/ethnicity				
White		Reference		
Black	1.81	1.34	2.44	<0.001
Hispanic	1.11	0.77	1.61	0.580
Asian or Pacific Islander	1.43	0.64	3.18	0.379
Native American	1.57	0.65	3.78	0.315
Other/unknown	1.14	0.77	1.69	0.516
Payer				
Private		Reference		
Public	1.13	0.87	1.45	0.363
Self-pay or no charge	1.08	0.76	1.54	0.653
Other/unknown	1.04	0.62	1.77	0.874
Urbanicity				
Nonurban areas	0.95	0.67	1.33	0.755
Urban areas		Reference		
Unknown	2.23	0.90	5.55	0.083
Household income				
Quartile 1	0.91	0.61	1.37	0.657
Quartile 2	1.04	0.69	1.57	0.856
Quartile 3	1.03	0.67	1.57	0.904
Quartile 4		Reference		
Unknown	1.36	0.66	2.81	0.403
Region of hospital				
Northeast		Reference		
Midwest	0.99	0.65	1.51	0.976
South	1.03	0.71	1.50	0.861
West	0.77	0.50	1.20	0.246
Ownership				
Public	0.75	0.56	1.02	0.067
Private		Reference		
Bed size				
Small	0.61	0.41	0.89	0.010
Medium	1.06	0.81	1.40	0.660
Large		Reference		
Unknown	1.22	0.61	2.44	0.579
Year				
2006		Reference		
2009	1.56	1.09	2.22	0.015
2012	2.02	1.39	2.95	<0.001
2019	0.96	0.66	1.40	0.826

HHS = hyperosmolar hyperglycemic state, DKA = diabetic ketoacidosis, CI = confidence interval.

**Table 3:** Severity of illness and mortality.

	HHS		DKA		*P* value	Total	
	*N*	(%)	*N*	(%)		*N*	(%)
Severity of illness					<0.001		
No class	≤10	0.0	≤10	0.0		≤10	0.0
Minor	≤10	0.5	28	0.3		30.3	0.3
Moderate	181	32.3	7,095	84.4		7,276	81.2
Major	306	54.6	1,042	12.4		1,348	15.0
Extreme	70	12.6	235	2.8		305	3.4
Mortality					0.0001		
Died	≤10	1.8	28	0.3		38	0.4

HHS = hyperosmolar hyperglycemic state, DKA = diabetic ketoacidosis.

**Table 4:** Length of stay (LOS) and admission charge.

	HHS	95% confidence interval	DKA	95% confidence interval
LOS (days)	3.94	3.12–4.75	3.16	3.07–3.26
Charge (2019 dollars)	$35,034.20	$26,615.58–$43,452.83	$27,008.10	$25,845.72–$28,170.48

HHS = hyperosmolar hyperglycemic state, DKA = diabetic ketoacidosis.

## Data Availability

The data used to support the findings of this study are available from the corresponding author upon request.
